# An Unbalanced Synaptic Transmission: Cause or Consequence of the Amyloid Oligomers Neurotoxicity?

**DOI:** 10.3390/ijms22115991

**Published:** 2021-06-01

**Authors:** Miriam Sciaccaluga, Alfredo Megaro, Giovanni Bellomo, Gabriele Ruffolo, Michele Romoli, Eleonora Palma, Cinzia Costa

**Affiliations:** 1 Neurology Clinic, Department of Medicine and Surgery, University of Perugia, Santa Maria della Misericordia Hospital, 06132 Perugia, Italy; alfredomegaro@gmail.com (A.M.); giovanni.bellomo@unipg.it (G.B.); 2 Department of Physiology and Pharmacology, Istituto Pasteur—Fondazione Cenci Bolognetti, University of Rome Sapienza, 00185 Rome, Italy; gabriele.ruffolo@uniroma1.it (G.R.); eleonora.palma@uniroma1.it (E.P.); 3 IRCCS San Raffaele Pisana, 00166 Rome, Italy; 4 Neurology Unit, Rimini “Infermi” Hospital—AUSL Romagna, 47923 Rimini, Italy; romoli.mic@gmail.com

**Keywords:** Aβ oligomers, hyperexcitability, excitatory/inhibitory unbalance, synaptic plasticity, network dysfunction, neurotoxicity, calcium homeostasis

## Abstract

Amyloid-β (Aβ) 1-40 and 1-42 peptides are key mediators of synaptic and cognitive dysfunction in Alzheimer’s disease (AD). Whereas in AD, Aβ is found to act as a pro-epileptogenic factor even before plaque formation, amyloid pathology has been detected among patients with epilepsy with increased risk of developing AD. Among Aβ aggregated species, soluble oligomers are suggested to be responsible for most of Aβ’s toxic effects. Aβ oligomers exert extracellular and intracellular toxicity through different mechanisms, including interaction with membrane receptors and the formation of ion-permeable channels in cellular membranes. These damages, linked to an unbalance between excitatory and inhibitory neurotransmission, often result in neuronal hyperexcitability and neural circuit dysfunction, which in turn increase Aβ deposition and facilitate neurodegeneration, resulting in an Aβ-driven vicious loop. In this review, we summarize the most representative literature on the effects that oligomeric Aβ induces on synaptic dysfunction and network disorganization.

## 1. Introduction

Amyloid-β (Aβ) 1-40 and 1-42 peptides are major actors in the pathophysiology of several neurodegenerative diseases. Initially sequenced from the meningeal blood vessels of patients with Alzheimer’s disease (AD) more than 30 years ago [1], they were rapidly recognized as the main component of senile plaques, which is a typical hallmark of AD. The subsequent cloning of the β-amyloid precursor protein (APP) allowed the identification of several mutations associated with AD and the profiling of biochemical abnormalities due to specific APP mutations [2]. The presence of senile plaques in AD brain tissue and the demonstration that insoluble fibrillar aggregates are neurotoxic in vivo and in vitro [3] contributed for years to support the “amyloid hypothesis” [2], according to which fibril accumulation *per se* underlies neuronal dysfunction in AD. Although the amyloid hypothesis provides a broad-spectrum explanation of AD pathogenesis, several observations obtained in both patients and experimental models do not completely fit with the hypothesis. Indeed, the local extent of neurodegeneration, as well as the severity of cognitive impairment, poorly correlate with the number of senile plaques [4]. Other studies demonstrate a strong correlation between the levels of soluble Aβ oligomers and the extent of synaptic deficit and the consequent severity of cognitive impairment [5,6]. However, all these findings about the bioactivity of small soluble oligomers do not rule out completely a role of amyloid plaques in the progressive neurodegeneration. Indeed, the presence of soluble/diffusible Aβ oligomers immediately surrounding the plaques is closely associated with local dendritic spine loss [7] and neuritic dystrophy [8], thus suggesting that plaques may serve as local inert reservoirs of these smaller neurotoxic peptides, that, during the process of fibrillogenesis [9], can diffuse away and cause injury to surrounding neurons. This latter hypothesis might explain at least in part the lack of correlation between amyloid plaques and memory impairment or cellular dysfunction. Indeed, soluble Aβ oligomers have demonstrated the ability to inhibit several critical neuronal activities and to impair hippocampal synaptic plasticity and memory already in the pre-plaque stage [10,11,12,13,14].

It has also been proposed that Aβ oligomers are able to interfere with homeostatic synaptic plasticity (HSP) and synaptic scaling mechanisms, with neurons differently adapting their synaptic properties in the presence of Aβ [15,16,17,18,19]. As a matter of fact, HSP has a critical role in the maintenance of neuronal function within a physiological range; thus, an impairment of these mechanisms leads to a destabilization in synaptic and neural circuit activity and potentially to an increased excitability of local neural networks that can constitute the substrate for an epileptiform activity. Epidemiologic studies indicate a close association between AD and incidence of epileptic seizures [20,21]. Interestingly, epileptiform activity is particularly high during the early stages of the disease or in younger AD patients [21,22]. Although the underlying mechanism of epileptic seizures in AD remains to be elucidated, this evidence suggests that cognitive decline and seizures activity may share common mechanisms. Studies performed in AD transgenic mice indicate that elevated levels of Aβ are able to elicit epileptiform activity and seizures, even at early stages of the disease, before evidence of neuronal loss [17,23,24]. All these evidences not only support the hypothesis that seizures may be the expression of pathophysiological processes similar to those responsible for cognitive decline, but also the possibility that aberrant excitatory neuronal activity may represent a primary upstream mechanism contributing to cognitive deficits in AD.

In this review, we will provide an overview of the possible mechanisms by which the pathological accumulation of oligomeric Aβ induces early synaptic dysfunction and network disorganization, ultimately leading to cognitive impairment. The identification of the affected circuits may help pave the way for the development of novel specific therapeutic strategies.

## 2. Molecular Mechanisms of Soluble Aβ Oligomers Formation and Toxicity

Aβ fibrillary aggregates are the major constituents of amyloid plaques in AD brains. Although Aβ1-40 is the most common isoform, Aβ1-42 is widely recognized as the most toxic species and the most prone to aggregation. The main difference between the two peptides is the presence of alanine 42, which confers to Aβ1-42 the possibility to form extra salt bridge between lysine 28 and alanine 42 during its aggregation kinetics. Aβ spontaneous aggregation is generally schematized as an initial “nucleation” of soluble oligomers from monomers and their subsequent conversion into protofibrils and fibrils [25]. The Aβ peptides are natively secreted in a monomeric unfolded state lacking a stable secondary structure. However, both Aβ1-40 and Aβ1-42 monomers tend to have an α-helix conformation, which is stabilized by particular ligands and/or microenvironments [26]. The transition from α-helix to β-sheet, and/or to a hybrid conformation called α-sheet [27], characterizes oligomers’ formation and their toxicity [26,28]. The structure of the transient oligomeric species might be different between two isoforms [29], with Aβ1-42 oligomers being more fibril-like (characterized by the typical cross-β-sheet motifs of fibrils), and the ones of Aβ1-40 being more globular and amorphous. In general, Aβ oligomers have variable molecular weight, with structural polymorphism also in similarly sized species. The heterogeneity among Aβ1-40 and Aβ1-42 oligomers not only accounts for their biological and structural diversity and for the complexity of AD pathology, but it also has considerably complicated their structural characterization and the elucidation of the atomic resolution structure [5,6,30,31,32]. Some structural data on Aβ oligomers have been obtained by transmission electron microscopy, atomic force microscopy, hydrogen/deuterium exchange, and fluorescence spectroscopy [33,34]. Most high-molecular-weight Aβ oligomers are roughly globular in shape or are annular with a pore or ring shape niche that encloses water [35,36,37,38]. The structure of these annular aggregates resembles that of pore-forming toxins, thus suggesting that they may have a potential to perturb the integrity of phospholipid bilayer membranes [39]. To this extent, Aβ can move between the interior of the cell and the extracellular space, and it can accumulate within living cells as well as in extracellular spaces [40,41]. 

Aβ oligomers express their toxicity through three main mechanisms: (1) by direct interaction with membrane receptors; (2) by damaging cellular/mitochondrial membranes; and (3) by interfering with vesicles trafficking and protein degradation mechanisms. In this review, we will mainly focus on the first two mechanisms, since interactions with membrane and membrane receptors can actively affect neuron transmission and excitability. The most representative studies linked to these effects induced by Aβ oligomers are summarized in Table 1.

### 2.1. Aβ Oligomers and Membrane Receptors

Several studies indicate that soluble globular Aβ oligomers (10–20 nm diameter, 200–300 kDa molecular mass) can be formed in the presence of GM1 ganglioside on the cell membrane [63,64]. Extracellular Aβ oligomers can bind several receptors on the cell surface, such as N-methyl-D-aspartate receptor (NMDAR), nerve growth factor receptor (NGFR), insulin receptor (IR), and Frizzled (Fz) receptor, leading to their functional disruption [61] or to an abnormal activation of downstream signaling pathways. Aβ oligomers induce NGFR -mediated neuronal death through the p75 neurotrophin receptor, which is a member of the tumor necrosis factor receptor superfamily [59,65]. For what concerns the IR, it has been reported that upon the binding with Aβ oligomers, insulin signaling is disrupted (thus suggesting that insulin resistance in AD brain is a response to the oligomers), and the receptor undergoes a redistribution on the cell surface with a consequent substantial loss of neuronal surface IRs, specifically on dendrites [55]. It has also been seen that Aβ oligomers are able to bind Fz receptors, producing the inhibition of Wnt signaling [47], which may result in intracellular tau phosphorylation and aggregation [66]. Several experimental evidences, obtained by both in vitro and in vivo studies, suggest that Aβ impairs NMDA-dependent long term potentiation (LTP) induction in the hippocampal CA1 and dentate gyrus (DG) by specifically interfering with major NMDAR downstream signaling pathways but also by disrupting the dynamic balance between protein kinase and phosphatase and by promoting the formation of reactive oxygen species (ROS) through a mechanism requiring NMDAR activation [58,67,68].

### 2.2. Aβ Oligomers Interaction with Cellular Membranes

One of the main molecular mechanisms proposed to explain neurotoxicity induced by Aβ is pore formation in membranes and the disruption of calcium homeostasis. In 1993, Arispe and coauthors demonstrated for the first time that Aβ peptides can be incorporated into artificial lipid bilayers, where they are able to form cation-selective channels [44,45]. Since then, evidence have been accumulated in favor of the potential of certain peptides, especially Aβ1-42, to form cation channels in neurons [53,69], oocytes, and endothelial cells [70,71,72,73,74]. Aβ1-40 and Aβ1-42 channels are calcium and zinc permeable [42,43,71,75], and they can profoundly disrupt ionic homeostasis. Aβ1-42 but not Aβ1-40 oligomers also form non-selective cation channels in cellular membranes, which is particularly detrimental for cell signaling [53]. Indeed, even small depolarizations of the membrane potential, due to increased membrane conductivity, could alter firing properties and lead to neural dysfunction. Since Aβ channels are large, cation-permeable (although poorly selective), and with a long lifetime [76,77], they likely affect the membrane potential generated by K^+^ selective channels in neurons. Moreover, Ca^2+^ influx can lead to aberrant signaling, altered neurotransmitter release and excitability due to the inhibition of presynaptic function as a consequence of calcium-dependent vesicular depletion [46], and it can even trigger apoptosis. All this evidence led to the formulation of the so-called “channel hypothesis”, according to which Aβ peptides damage neurons by forming ion channels [76]. 

### 2.3. Intracellular Aβ Oligomers Affecting Neural Transmission and Excitability

Intraneuronal accumulation of Aβ has been found in AD patients as well as in animal models or in cultured cells [78,79,80,81,82], and it has been reported to occur prior to amyloid plaques deposition and to be deeply involved in synaptic dysfunction [83,84,85,86]. In contrast to extracellular mature fibrils, extracellular soluble oligomers can be efficiently internalized by glial and neuronal cells [68,87,88]. However, it is well known that Aβ can be produced intracellularly within the endoplasmic reticulum (ER) and the trans-Golgi network system along the secretory pathway [89,90,91], and it has been reported to localize also in endosomal, lysosomal [90], and mitochondrial membranes [92,93,94]. The accumulation of Aβ at mitochondria levels leads to an impairment of respiratory chain complex III and IV activity [48], which can be at the basis of the mitochondrial deficits observed both in patients and in mouse models of AD [95]. As it is observed for cellular membranes, Aβ oligomers may damage mitochondrial membranes and perturb Ca^2+^ homeostasis [54]. Indeed, energetic failure and an increased production of ROS has been widely documented in AD [96]. Excessive production of ROS by dysfunctional mitochondria can activate pathological signaling cascades, affecting multiple neuronal functions [97] and, among them, abnormal processing of APP and the generation of toxic Aβ peptides [93]. In turn, these exacerbate mitochondria dysfunction and energy failure, further enhancing the production of ROS and Aβ [98,99], which becomes a vicious loop finally leading to cognitive impairment [96,100,101]. Interestingly, Aβ oligomers also interfere with mitochondrial bidirectional axonal trafficking [102,103]. The mobility partner of mitochondria in neurons is complex, finely modulated, and characterized by frequent changes in directions and by the capability to leave mitochondria stationary or to quickly mobilize them in the areas of greatest energy demand, according to physiological changes [104,105,106]. At synapses, mitochondrial energy supply is fundamental for several neuronal functions, including the mobilization of synaptic vesicles and the generation of membrane potentials [107,108,109]. Moreover, due to the elevated capability to sequester calcium, mitochondria play a pivotal role in maintaining calcium homeostasis at synapses by buffering the excess of intracellular calcium and releasing it after stimulation [110,111,112]. This mechanism prolongs residual calcium levels [113] and allows the modulation of synaptic transmission [110,111,112,114] and of short-term synaptic plasticity [115,116]. In this scenario, the inhibition of mitochondria trafficking and dynamics may contribute to synaptic impairment and consequent cognitive deficit.

All this evidence suggests that extracellular and intracellular oligomers exert their toxicity through different mechanisms, but more studies are needed to elucidate these mechanisms in relation to pathology. 

## 3. Calcium Homeostasis and Oligomer-Mediated Synaptotoxicity

The mechanism underlying oligomer synaptotoxicity appears to be closely related to impairment in calcium homeostasis [117]. In the central nervous system (CNS), calcium plays a pivotal role in neuronal excitability, in evoking LTP or long-term depression (LTD) and in higher cognitive functions [118,119,120]. Due to its fundamental role in neuronal physiology, intracellular calcium concentration ([Ca^2+^]_i_) is tightly modulated by a complex interplay between Ca^2+^ influx, Ca^2+^ efflux, intra-organelle sequestration, and buffering [117]. Calcium dynamics are deeply influenced by APP metabolism, since almost all APP hydrolysis products are able to modulate calcium signaling and dynamics with both stabilizing or destabilizing outcome [117]. In particular, while secreted APP is generally neuroprotective and normalizes cytosolic calcium levels [121], Aβ oligomers increase intracellular calcium [122,123], leading to Ca^2+^ homeostasis dysregulation. This effect is mainly due to the capability of Aβ oligomers to form cation-selective channels on plasma membrane [42,43,75,76,77], but also, the interaction of Aβ oligomers with membrane receptors, such as NMDAR, metabotropic glutamate receptor 5 (mGluR5), and α7-nicotinic acetylcholine receptor (nAChR), may play a role [60,124]. In addition to altered neurotransmitter release and excitability [46], excessive intracellular Ca^2+^ levels trigger aberrant signaling cascades, adversely affect a plethora of cellular enzymes (such as proteases, phospholipases, kinases, and phosphatases), induce specific cytoskeletal rearrangements, and trigger apoptosis [117]. Among other signaling pathways, calcium increase triggers the activation of the Ca^2+^/calmodulin-dependent phosphatase calcineurin and glycogen synthase kinase 3β, which, in turn, can induce hyperphosphorylation of tau and cause transport dysregulation in both axons and dendrites also impairing the transport of brain-derived neurotrophic factor [125].

It has been reported that elevated levels of Aβ block neuronal glutamate uptake in the synaptic cleft [57], with a consequent glutamate spillover and activation of extrasynaptic NMDAR and mGluRs, which contribute to increased Ca^2+^ influx and release from ER. Experimental evidence demonstrate that Ca^2+^ that enters the cytoplasm through NMDAR activation has more rapid access to mitochondria [126]. Thanks to their ability to accumulate an enormous amount of calcium, mithocondria play a key role in orchestrating Ca^2+^-dependent responses in neurons: the controlled integration among mitochondrial Ca^2+^ uptake, sequestration, and release is essential in modulating and interpreting neuronal responses that range from gene transcription to cell death. Under physiological conditions, the rapid mitochondrial Ca^2+^ uptake may contribute to promote mitochondrial respiration and energy production and help prevent an excessive rise in cytosolic Ca^2+^ and shape the [Ca^2+^]_i_ response after NMDAR stimulation. However, this fast mitochondrial Ca^2+^ uptake induced by NMDA may allow excessive mitochondrial Ca^2+^ accumulation, making mitochondria more susceptible to Ca^2+^-mediated injury and thus converting a protective mechanism into a toxic mechanism during excessive or prolonged NMDA receptor activation. A loss of intracellular Ca^2+^ homeostasis causes mitochondrial Ca^2+^ overload and mitochondrial dysfunction with the production of reactive oxygen species inducing membrane-lipid peroxidation, failure in the respiratory chain and in bioenergetics, increased mitochondria permeability and activation of Ca^2+^-dependent proteases such as calpains, finally leading to neuronal cell death [127,128,129]. Calcium dysregulation at mitochondrial levels also interferes with mitophagy, which is an essential process to remove damaged mitochondria, which plays a key role in the adjustment of the functional integrity of the mitochondrial network and cell survival [130,131]. This further contributes to the generation of Aβ oligomers and AD progression, creating a vicious circle between Aβ and mitochondria dysfunction [132,133]. Indeed, calcium is able to modulate APP processing and Aβ production and/or release in a different manner, depending on whether the source of cytosolic calcium is represented by calcium permeable ion channels on the cell membrane, intracellular stores, inositol-1,4,5-trisphosphate, or ryanodine-sensitive pools [117].

Cytoplasmic Ca^2+^ also plays a role in modulating mitochondrial dynamics. In particular, elevated intracellular Ca^2+^ levels, due to synaptic activity and voltage-dependent Ca^2+^ channels or NMDAR activation, inhibit mitochondrial motility [134,135,136,137]. In addition to cytosolic Ca^2+^, a recent study suggests that mitochondrial matrix Ca^2+^ content is able to modulate mitochondrial transport in hippocampal neurons as well by a not completely elucidated mechanism involving the Ca^2+^ sensor MIRO [136]. Moreover, Ca^2+^ influx facilitates mitochondrial fragmentation by Ca^2+^/calmodulin-dependent protein kinase I activation and subsequent phosphorylation of the fission protein dynamin-related protein 1, which in turn increases its interaction with mitochondrial fission 1 protein, thus enhancing the fragmentation process [138]. Due to the role of mitochondria and mitochondrial motility in supporting synaptic activity and Ca^2+^ buffering, these Ca^2+^-induced alterations could contribute to synaptic dysfunction, neuronal loss, and memory deterioration.

## 4. Impairment of Synaptic Excitability, Transmission, and Plasticity

The attempt to correlate the alteration of synaptic plasticity, hippocampal-dependent memory deficits, and plaque formation revealed that alterations of dendritic spine density, impaired LTP, and behavioral deficits occur months before plaque deposition [139,140], but the temporal and causal links among LTP alterations, formation of different amyloid peptides, and deposition of plaques still remain to be elucidated.

To date, despite several reports indicating that the accumulation of cerebral Aβ peptide is essential for developing synaptic and cognitive deficits, the initial mechanisms underlying early Aβ-mediated synaptic dysfunctions, as well as the physiological roles of Aβ, remain largely unknown. Several experimental studies suggest that early Aβ-induced pathology is associated with neuronal excitability that arises in a pre-plaque stage [62,141,142,143,144,145], which is in agreement with the idea that epileptic activity might be prodromal to dementia [62,146,147]. Transgenic mice expressing mutated forms of the APP, which is associated with familial AD, display age-dependent dysfunctions before plaque deposition is detectable [62,145]. In this model, high levels of Aβ oligomers are able to elicit epileptiform activity and seizures at early stages of the disease process and in the absence of evident neuronal loss [23]. Although several studies demonstrate that brain extracts from both AD patients or TG2576 transgenic mouse models are able to induce plaque deposition [148,149], the role of soluble Aβ oligomers in plaque deposition is still a matter of debate. In this respect, it has been proposed that Aβ oligomers can be classified into toxic and non-toxic [150,151]. According to this hypothesis, toxic Aβ oligomers (Type 1) are associated with memory impairment and unrelated with amyloid plaques, while non-toxic Aβ oligomers (Type 2) are spatially and temporally related to plaques but not with memory impairment [151]. Studies, performed both in vitro and in vivo, demonstrated that Aβ oligomers are able to impair synaptic transmission at both the presynaptic and post-synaptic level in a dose- and assembly-dependent manner [16,152]. High levels of Aβ cause synaptic loss by impairing glutamatergic synaptic transmission [11,83,153,154]. Interestingly, neuronal activity has been reported to modulate Aβ production and secretion in a positive manner at the presynaptic level by mechanisms involving clathrin-mediated endocytosis, proteolytic cleavage of APP, and Aβ release [154,155] in both pathological and physiological conditions [155,156]. This finding supported the hypothesis that APP and Aβ take part in a feedback loop controlling neuronal excitability [154] and led to the formulation of a model in which intermediate levels of Aβ enhance pre-synaptic facilitation, while abnormal levels of Aβ impair synaptic plasticity by inducing post-synaptic depression (high levels) or by reducing pre-synaptic efficacy (low levels) [16,144]. According to this model, while small increases of Aβ in the physiological range result in synaptic potentiation [157,158], abnormally high levels result in post-synaptic depression and loss of dendritic spines [57,61,154,159]. In this view, this physiological negative feedback regulator is put in overdrive by pathological elevations of Aβ levels, with a consequent suppression of excitatory post-synaptic activity.

On the other hand, it has been reported that inhibition of Aβ degradation, leading to a small increase in endogenous Aβ levels, enhances spontaneous excitatory post-synaptic currents by increasing synaptic vesicles release probability, thus suggesting that Aβ can play a role as a positive regulator at the presynaptic level [158]. This presynaptic facilitation was lower for those neurons with higher firing rates. This implies that Aβ-mediated presynaptic facilitation occurs in neurons with low activity. Indeed, in agreement with this view, picomolar Aβ positively modulates synaptic plasticity in the hippocampus by a presynaptic α7-nAChR-dependent mechanism [157], while higher concentrations (in the nanomolar range) cause synaptic depression. The mechanism underlying this positive modulatory role of Aβ in neurotransmission is probably a positive feedback loop in which the increased [Ca^2+^]_i_ downstream from the direct activation of presynaptic α7-nAChR by Aβ [160] promotes Aβ secretion, as proven by the demonstration that inhibition or removal of α7-nAChR reduced Aβ secretion, thus blocking Aβ-induced synaptic facilitation [156]. Overall, this evidence indicates that an optimal concentration of extracellular Aβ is fundamental for Aβ-induced presynaptic facilitation, with higher or lower concentrations impairing synaptic transmission [158]. This phenomenon has been clearly represented by a bell-shaped relationship between extracellular concentrations of Aβ and synaptic transmission, with intermediate and low levels respectively potentiating or impairing presynaptic transmission and high levels depressing postsynaptic transmission [16]. 

Excitatory synaptic transmission is tightly modulated by the number of active NMDARs and α-amino-3-hydroxy-5-methyl-4-isoxazolepropionic acid receptors (AMPARs) at the synapse. These glutamate-gated ion channels are essential mediators of synaptic plasticity, being able to convert specific networks of neuronal activity into the long-term changes in synapse structure and activity that underlie high cognitive functions [161]. Functional NMDARs are tetrameric complexes of several homologous subunits (GluN1, GluN2A–GluN2D, GluN3A, and GluN3B) that assemble with a plastic stoichiometry, resulting in a large number of receptor subtypes with distinct biophysical, pharmacological, and signaling properties [161]. NMDAR activation plays a central role in the outcome of synaptic plasticity processes, being able to trigger either LTP or LTD, depending on the extent of the resultant increase in [Ca^2+^]_i_ at the post-synaptic level and the downstream activation of specific intracellular signaling cascades [162]. Indeed, high calcium levels are necessary for LTP induction, which also promotes the recruitment of AMPARs and the growth of dendritic spines. Conversely, low calcium rise induces LTD, together with extra-synaptic NMDAR activation, receptor internalization at the synapses, spine shrinkage, and synaptic loss [162]. Elevated levels of Aβ, in the pathological range, have been reported to impair LTP and enhance LTD, inducing synaptic loss [11] by mechanisms involving either glutamate receptor trafficking [57,61,163] or the activation of NMDAR downstream signaling cascade involved in LTD induction [159]. Moreover, elevated levels of Aβ have been demonstrated the ability to block neuronal glutamate uptake in the synaptic cleft [57], with a consequent desensitization of activated NMDAR, glutamate spillover, and activation of extra-synaptic GluN2B-enriched NMDARs and mGluRs, resulting in LTD induction [164,165]. In this view, the facilitation of LTD and inhibition of LTP by Aβ may arise from an initial enhanced activity of synaptic NMDAR followed by desensitization, internalization, and activation of GluN2B-enriched extra-synaptic glutamate receptors. The NMDARs subunit composition is involved in the fine control of the post-synaptic dynamics of Ca^2+^ and, consequently, in the regulation of the direction of synaptic plasticity, since Ca^2+^ influx through NMDARs is critical for the induction of NMDAR-dependent LTP and LTD [120,166]. Indeed, GluN2B-containing NMDARs bind with Ca^2+^/calmodulin-dependent protein kinase II (CaMKII) with a higher affinity with respect to those containing GluN2A subunits [167]. Due to its ability to bind to different NMDAR subunits with different affinity, CaMKII constitutes a key mediator in the control of Hebbian synaptic plasticity [167], which functions as a positive feedback mechanism that progressively modifies network properties and eventually leads to unstable excitation [168]. In physiological conditions, HSP is able to counteract the destabilizing effects of Hebbian plasticity by a negative feedback control mechanism that allows a compensatory refinement of synaptic strength, thus maintaining the stability of network activity [169,170,171,172]. Thus, an impairment of HSP, and in particular of metaplasticity (a form of HSP that controls the induction threshold of LTP and LTD) can cause aberrant Hebbian plasticity, leading to pathological synaptic potentiation or depression [173]. Interestingly, Aβ-induced aberrant hyperexcitability has been reported in cortical and hippocampal neuronal networks of patients and mouse models of AD [16,18,20,141,174,175]. On the other hand, epileptiform activity in the hippocampus has been demonstrated to enhance the levels of Aβ [154,155,176], thus generating a positive feedback loop between hyperexcitability and Aβ production, finally favoring LTP inhibition and LTD induction. In this respect, another crucial mechanism for many forms of synaptic plasticity and remodeling is the activity-dependent AMPAR trafficking [120,177]. AMPARs are tetrameric assemblies of dimers of four different subunits (GluAR1–GluR4). The presence of GluR2 subunit confers Ca^2+^ impermeability and influences channel kinetics and conductance as well as AMPAR assembly and trafficking at synapses [161]. AMPARs mediate the majority of fast excitatory synaptic transmission by ensuring rapid responses to synaptic released glutamate. Depending on the frequency of the synaptic activity, AMPARs are either inserted or removed from synapses, resulting in the potentiation or depression of synaptic transmission, respectively [120]. One of the most well-studied pathophysiological phenomena that involves AMPARs is their oligomer-induced internalization [163], for which different molecular mechanisms have been hypothesized [178]. For instance, AMPAR’s GluR3 subunit was found to be involved in receptor internalization in the early phases of AD, leading to the onset of memory deficits in a mouse model of disease [179]. Furthermore, GluR1 ubiquitination may also lead to receptor internalization following exposure to Aβ peptides [180]. On the other hand, the insertion in membranes of AMPARs can be induced by CaMKII, which can be inhibited by Aβ accumulation, leading to a disturbed synaptic trafficking [181].

Since alteration in the number of AMPARs localized at synapses underlies changes in the strength of synaptic transmission [177], the insertion and removal of synaptic AMPARs is a process finely regulated by the phosphorylation of AMPARs GluR1 at Ser-845 and Ser-831 [182,183,184]. In particular, the Ser-845 phosphorylation of GluR1 subunits mediates the insertion of Ca^2+^-permeable (GluR1-containing) AMPARs at synapses during inactivity-induced synaptic scaling in cultured dissociated cortical neurons [185] as well as in vivo in the visual cortex [186,187,188,189] and in spinal cord [190,191]. These studies also indicate that the expression of HSP under chronic suppression of neuronal activity occurs through an increased insertion of AMPARs at synapses, culminating in an upscaling of AMPAR-mediated miniature post-synaptic currents. It has been reported that local injection of Aβ in vivo, in the visual cortex, results in an up-regulation of AMPAR-mediated synaptic currents and in an aberrant cell-surface expression of calcium-permeable AMPARs, which are required for the initiation of homeostatic plasticity but not for its maintenance [15]. Thus, this enhanced and prolonged expression of Ca^2+^-permeable AMPARs appears to be the cause of an aberrant over-scaling of synaptic strength. Therefore, the presence of Aβ can trigger a saturation of neuronal synaptic response with a consequent destabilization of neural network and impairment of information processing, finally leading to cognitive deficits. Moreover, Aβ-induced synaptic over-scaling can likely increase the overall excitability of the local neural network that can constitute the substrate for the epileptiform activity associated with the early phases of AD progression [21,22]. The exposure of rodent hippocampal neurons and slides to Aβ oligomers, as well as intracisternal injection of Aβ, is able to elicit pro-epileptogenic changes and to facilitate seizure and synaptic coupling [192,193,194], while in the Tg2576 mouse model, which is characterized by a progressive increase in Aβ production and deposition, Aβ oligomers have been demonstrated to affect intrinsic and extrinsic neuronal properties impairing dentate gyrus transmission and lowering the hippocampal seizure threshold [62,195,196]. The enhanced epileptic activity observed in the DG of both this mouse model and of oligomer-treated slices appeared to be related with a dysfunction in D1-dopamine(DA) receptor transmission [62]. Indeed, the surface expression of D1 receptors was increased in both experimental models, although with different mechanisms, and the epileptic-like activity was facilitated by receptor stimulation and blocked by D1 receptor antagonists [62], suggesting a DA-mediated epileptic susceptibility in both experimental paradigms. 

**Figure 1:**
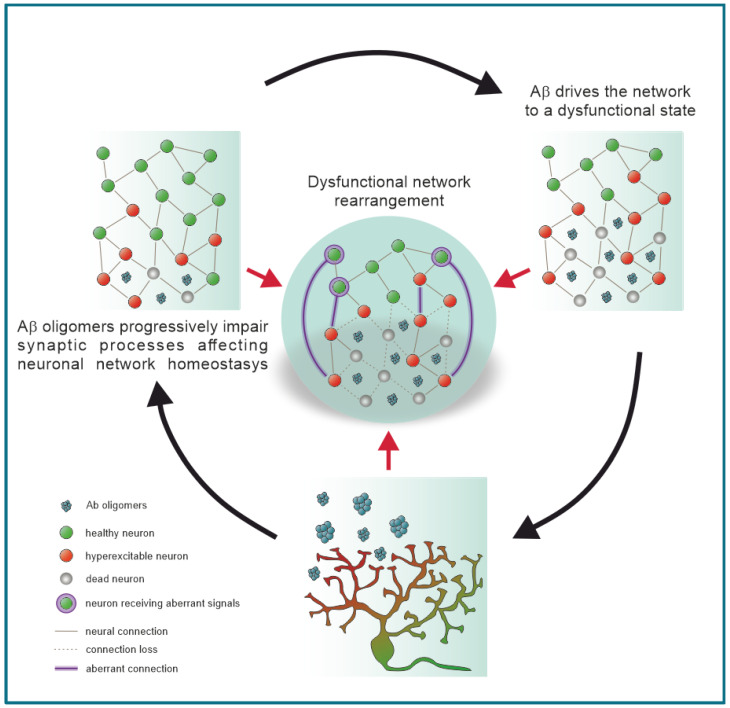
Aβ oligomers take part in a feedback loop influencing neuronal excitability, synaptic rearrangement, and neuronal death. Aβ oligomers-induced damages provoke the impairment of cellular homeostasis (bottom panel). The spreading of Aβ oligomers induces neural alterations, causing the emergence of aberrant hyperexcitability (left panel), subsequent cell death (right panel), physiological connection loss, synaptic rearrangement, and the formation of new non-physiological communication pathways (central panel) at the network level. Aβ-induced synaptic over-scaling can likely increase the overall excitability of local neural networks.

Aβ oligomers have been reported to be synaptotoxic [158,197,198] and to induce epileptic discharges before plaque deposition [62,199]. In fact, hippocampal neuron hyperactivity and spontaneous firing have been demonstrated during early stages of Aβ pathology in transgenic mice overexpressing mutant APP [19,141]. Morphological and functional alterations similar to those elicited by excitotoxic stimuli have been reported in three different models of APP transgenic mouse [17,200]. These alterations were associated with cortical and hippocampal non-convulsive seizures, thus indicating a possible causal link between Aβ accumulation and epileptogenesis. Taken together, all this evidence suggests that Aβ might initially induce a hyperactive neural phenotype, which over time will involve an increasing number of neurons, driving excitatory/inhibitory imbalance, synaptic impairment and epileptogenesis. In turn, all these processes increase Aβ deposition and facilitate neurodegeneration, resulting in an Aβ-driven vicious loop (Figure 1). Another possibility is that Aβ oligomers alter network functionality and plasticity by non-neuronal mechanisms. A relevant factor in enhancing neuronal excitability is represented by inflammation, which is a common feature of both AD and epilepsy [201]. In a recent report, Dejakaisaya and co-authors [202] hypothesized that the disruption of brain glutamate homeostasis, in which astrocytes play a key role, could constitute the link between AD and epilepsy. These authors speculate that the impairment in glutamate uptake is an early event occurring before plaque deposition and due to astrogliosis, which in turn enhances the susceptibility to epileptogenesis through the accumulation of extracellular glutamate and consequent excitotoxicity.

## 5. Oligomers and Alteration of Excitatory/Inhibitory (E/I) Neurotransmission

The impairment of brain function in AD both in patients and animal models of the disease can be related to a plethora of pathophysiological mechanisms. However, it is necessary to increase our knowledge on the unbalance between excitatory and inhibitory neurotransmission. In this regard, the Aβ oligomers may play an important role [193,203]. Indeed, E/I unbalance may be caused by the effect of Aβ oligomers on different neurotransmitter receptors. Substantial evidence supports the role of oligomers in the impairment of NMDA- and AMPA-mediated neurotransmission, which leads to an alteration of LTP and LTD, as reported above [9,61,204,205]. On the other hand, even if the GABAergic system has long been considered as relatively conserved in AD and hence spared by the Aβ-induced neurodegeneration [206], recent evidence points out that also inhibitory neurotransmission might play its role in this phenomenon. Gamma-Aminobutyric acid (GABA) is the main inhibitory neurotransmitter in CNS, binding to several subtypes of receptors: GABA_A_ receptors (GABA_A_Rs), GABA_B_ receptors (GABA_B_Rs), and GABA_C_ receptors (GABA_C_Rs). Between them, GABA_A_Rs possess a prominent role in neurotransmission. Indeed, these ionotropic pentameric receptors are responsible for both phasic (mainly α1β2γ2 receptors, the most common isoform according to the literature) and tonic inhibition (α4-α6- and δ-containing GABA_A_R) [207] and thus represent relevant pharmacological targets for neurological diseases. 

Several authors reported that Aβ oligomers may influence GABA_A_Rs trafficking and function [208,209]. Notably, it has been shown that Aβ1-42 can induce a downregulation of GABA_A_Rs in the rat somatosensory cortex [208] and Aβ40 can modulate the expression of the α6 GABA_A_Rs subunit [209]. On the other hand, the GABAergic tone can influence the detrimental effect of Aβ, since it has been shown that increasing GABAergic tone can prevent the Aβ-induced impairment of hippocampal LTP [193]. The interpretation of the literature is not straightforward, and sometimes, divergent results are reported but, nonetheless, a rather interesting study on mice hippocampal slices exposed to disease-relevant forms of Aβ, isolated from the AD brain, revealed that the net effect of Aβ toxicity may be an increase in excitability, coupled with a decrease of the efficiency of inhibitory transmission [210,211].

At a higher level of complexity in brain “micro-circuitry”, E/I unbalance may be determined by a dysfunction of GABAergic interneurons, since their activity may be affected by amyloid deposition mainly in the brain networks with high metabolic rates [212]. In addition, the diminished efficiency of these cells caused by oligomers accumulation may explain a disinhibition of glutamatergic neurons, resulting in an increased excitatory tone [211,213]. 

Therefore, it is not surprising that the brain hyperexcitability has been linked to the preclinical stages of AD, while it is replaced in the later stages of the pathology by a condition of hypoexcitability [144]. This has been confirmed by means of fMRI study of mild cognitive impairment patients, who showed a greater hippocampal activation than the controls, while the presumed AD patients showed a hippocampal and entorhinal hypoactivation and atrophy [214]. Interestingly, the earliness of this “state of hyperexcitability” may make it a suitable target for therapeutic intervention. To strengthen this notion, it has been demonstrated that treatment with the antiseizure medication levetiracetam is able to reduce cognitive impairment in mild AD individuals [215,216]. 

Another intriguing element for debate is the relationship between seizure-like activity and oligomers-induced neurodegeneration. It is now well documented that seizures in AD appear, depending on the different reports, from 10% to 64% of the cases [144,217]. It is also likely that in most cases, seizure-like activity in AD may be overseen, due to its subclinical nature [218]. Estimates and epidemiological data aside, which is the exact pathophysiology of these phenomena? A hint on the answer to this question may come from the observations that epileptiform activity precedes by many years the manifestation of cognitive impairment, and that AD cases that undergo cognitive decline more rapidly are often also affected by epileptic seizures [219]. Notably, seizures may not only be seen as a consequence of neurodegeneration induced by oligomers accumulation, but epileptic discharges may serve themselves as a facilitating factor for amyloid deposition, since amyloid burden was found to be significantly increased in a population of adult patients with childhood-onset epilepsy [220]. On the other side of the E/I scale, glutamate receptors’ dysfunctions have been associated with oligomer-induced alterations of neurotransmission [178,221]. The modulation of NMDAR by memantine has been reported to restore LTP in the DG of mice expressing the Swedish-Indiana APP mutation [222], and this effect was due to a normalization of the NMDA to AMPA ratio. The therapeutic effects of memantine, which is currently approved for the treatment of AD and other dementias, are mainly associated to its neuroprotective effects against excitotoxicity arising from NMDAR overactivity in pathological conditions [223]. However, the lack of beneficial effects in the early stage of AD led to the hypothesis that its action is not achieved only through neuroprotection but is rather the result of a correction of the E/I unbalance [224].

## 6. Conclusions

These data may suggest that oligomer-induced neurotoxicity undermines a correct balance between excitation and inhibition because it greatly enhances a form of “pathologic” and exaggerated excitatory neurotransmission [225] while impairing the functional dialogue between AMPA and NMDARs, leading to synapses maintenance and potentiation [221]. Additionally, oligomers may decrease the efficacy of inhibitory neurotransmission, thus worsening the E/I imbalance [193,206,208,209]. Even though the exact chain of events and cellular mechanisms leading to this scenario is not yet completely clear, some findings linking the restoration of the inhibitory function to the prevention of cognitive deficits in animal models of disease [226,227] clearly suggest that E/I disruption may be both a key pathophysiological mechanism and an innovative therapeutic target.

## Figures and Tables

**Table 1:** Summary of the principal evidence demonstrating Aβ oligomers-induced effects linked to neuronal transmission and excitability.

Type of Aβ	Interactions	Effects	Models	Ref.
Aβ1-40 oligomers	membranes	↑ Ca^2+^	GnRH neuronal cell line	[42]
			cultured endothelial cells	[43]
			bilayer membranes	[44,45]
		↓ neurotransmitter release	hippocampal neurons	[46]
	FzR	↓ Wnt/Fz signaling	N2A cells and L-cells	[47]
	mitochondria	↓ complex IV activity	APP Tg mice and human brain samples	[48]
Aβ1-42 oligomers	membranes	↑ Ca^2+^	lipid vesicles	[49]
			cultured endothelial cells	[43]
			SH-SY5Y cells, oocytes	[50,51]
			hippocampal neurons	[52]
		↓ axonal transport		
		↑ non-specific ionic flux	neuronal HEK293 membranes	[53]
		↓ mitochondrial membrane potential	hippocampal neurons	[54]
		↑ oxidative stress		
	IR	↓ activity of IR	hippocampal and cortical neurons	[55]
	mGluR/NMDAR	↑ Ca^2+^	hippocampal neurons	[56]
		↑ synaptic glutamate, LTD		[57]
	mGluR	↑ synaptic damage		[56]
	mitochondria	↓ mitochondrial membrane potential	APP Tg mice and human brain samples	[48]
		↑ oxidative stress		
	NMDAR		hippocampal neurons	[58]
	p75NTR	↑ NGF-mediated cell death	PC12 cells	[59]
	α7/α4β2nAChRs	↑ Ca^2+^	hippocampal neurons	[60]
			cortical neurons	[61]
		↓ surface AMPAR expression	hippocampal neurons	[60]
		↑ endocytosis of NMDAR	cortical neurons	[61]
	D1 DAR	↑ epileptic-like activity	APP Tg mice	[62]

Legend: FzR = Frizzled receptors, IR = insulin receptors, mGluR = metabotropic glutamate receptor, NMDAR = NMDA receptor, p75NTR = p75 neurotrophin receptor, nAChRs = nicotinic acetylcholine receptors, D1 DAR = D1 dopamine receptors, GnRH = gonadotropin-releasing hormone, AMPAR = AMPA receptors, APP = amyloid precursor protein, NGF = nerve growth factor.
